# Health Care Professionals’ Views on Using Remote Measurement Technology in Managing Central Nervous System Disorders: Qualitative Interview Study

**DOI:** 10.2196/17414

**Published:** 2020-07-24

**Authors:** Jacob A Andrews, Michael P Craven, Jennifer Jamnadas-Khoda, Alexandra R Lang, Richard Morriss, Chris Hollis, The RADAR-CNS Consortium

**Affiliations:** 1 Division of Psychiatry and Applied Psychology, Institute of Mental Health School of Medicine University of Nottingham Nottingham United Kingdom; 2 NIHR MindTech Medtech Co-operative Institute of Mental Health University of Nottingham Nottingham United Kingdom; 3 Bioengineering Research Group Faculty of Engineering University of Nottingham Nottingham United Kingdom; 4 Human Factors Research Group Faculty of Engineering University of Nottingham Nottingham United Kingdom; 5 NIHR Nottingham Biomedical Research Centre University of Nottingham Nottingham United Kingdom; 6 The RADAR-CNS Consortium London United Kingdom

**Keywords:** epilepsy, multiple sclerosis, depression, medical devices, barriers, health personnel, qualitative, mobile phones, mHealth, eHealth

## Abstract

**Background:**

Remote measurement technologies (RMT) can be used to collect data on a variety of bio-behavioral variables, which may improve the care of patients with central nervous system disorders. Although various studies have explored their potential, prior work has highlighted a knowledge gap in health care professionals’ (HCPs) perceptions of the value of RMT in clinical practice.

**Objective:**

This study aims to understand HCPs’ perspectives on using RMT in health care practice for the care of patients with depression, epilepsy, or multiple sclerosis (MS).

**Methods:**

Semistructured interviews were conducted with 26 multidisciplinary primary and secondary care HCPs who care for patients with epilepsy, depression, or MS. Interviews were transcribed verbatim and analyzed using thematic analysis.

**Results:**

A total of 8 main themes emerged from the analysis: (1) potential clinical value of RMT data; (2) when to use RMT in care pathways; (3) roles of health care staff who may use RMT data; (4) presentation and accessibility of data; (5) obstacles to successful use of RMT; (6) limits to the role of RMT; (7) empowering patients; and (8) considerations around alert-based systems.

**Conclusions:**

RMT could add value to the system of care for patients with central nervous system disorders by providing clinicians with graphic summaries of data in the patient record. Barriers of both technical and human nature should be considered when using these technologies, as should the limits to the benefits they can offer.

## Introduction

### Background

In a health care context, remote measurement technologies (RMT) can be used by health care professionals (HCPs) and clinical teams to collect data on a patient’s health or behavior and use this to inform clinical decision making. The benefits of RMT have been explored in the management of patients with cardiac conditions [1-3], early stage dementia [4], neurological disease [5], and attention deficit hyperactivity disorder [6] as well as in behavior change [7] and monitoring for indicators of sepsis [8] among others.

The European Union H2020 Remote Assessment of Disease and Relapse-Central Nervous System (RADAR-CNS) project [9] explores the use of RMT in the care of patients with epilepsy, depression, or multiple sclerosis (MS). These conditions were chosen for this project as exemplars of central nervous system disorders that are under-researched in relation to RMT. As part of this project, RADAR-base, a cloud-based platform, is being developed to explore the potential to receive data from patients’ RMT and to provide these data to HCPs with a view to informing clinical decision making [10]. This study is part of this project, aiming to understand the clinical utility of RMT in the care of patients with epilepsy, depression, or MS.

Previous literature has demonstrated the benefits of using RMT in health care practice. A 2013 literature review of the use of RMT in cardiology identified a number of studies demonstrating reduced hospital visits in terms of both emergency and routine appointments, as well as higher survival rates, in patients who were monitored using RMT [11]. Benefits were also found in patients’ relations with the care team, quality of life, and compliance with treatments. However, no quality appraisal was conducted in this narrative review.

Some studies have challenged these findings, suggesting little or no evidence of an effect of RMT on key outcomes [12,13]. Other work has highlighted a number of barriers to implementing RMT in health care practice. Erdmier et al [14] described a lack of regulatory control over wearables as well as a number of barriers to progress in implementation, including technical capability, erratic user (patient) behavior, and a lack of transparency from manufacturers. A patient and HCP-led priority setting exercise in the field of digital mental health highlighted the need to explore the impact of removing face-to-face human interaction in care pathways for mental health conditions, and raised issues of safety, effectiveness, evaluation, and inequalities [15]. These issues apply equally to the use of RMT.

### Objectives

Authors of prior work in the area of RMT highlighted a need for research to investigate the value to HCPs of implementing RMT [13]. Davis et al [16] conducted a systematic review of health care staffs’ views on the use of RMT in clinical practice and included 15 relevant studies. They concluded that “there is a critical need to engage end-users in the development and implementation of RMT” and highlighted that the evidence base in this area is small. This paper seeks to address these points by exploring HCPs’ perspectives on the implementation of RMT in three central nervous system disorders.

## Methods

### Aim

This study aims to understand the perspectives of HCPs on the use of RMT in health care practice for the care of patients with depression, epilepsy, or MS.

### Recruitment

We purposively recruited a sample of 26 HCPs, with the intention of covering multiple clinical roles (with representation from medical, nursing, and allied health professionals). Participants were all working in the National Health Service (NHS) in England in the care of patients with epilepsy, depression, or MS or a combination of these conditions. Participants were contacted through the professional networks of the research team members.

### Procedure

A semistructured interview approach was used, with interviews lasting from 16 to 56 min (mean 30 min). An interview schedule was used to guide questioning, with ad hoc follow-up questions used to further explore salient points. Participants gave informed consent and were incentivized with a £15 (US $18) charity donation.

A total of 23 interviews were conducted one to one, whereas one interview was conducted with three participants together. Moreover, 13 interviews were conducted by phone, whereas 11 were conducted in person. All interviews were recorded from start to finish using a voice recorder. The study was approved by the University of Nottingham School of Medicine research ethics committee (ref 277-1802).

### Analysis

Interviews were transcribed verbatim and analyzed using thematic analysis [17]. Data were coded and themed (by JA) using NVivo 12 (QSR International). Initial codes and themes were discussed within the research team and were iteratively renamed and reformed throughout the analysis process. No new themes emerged when the last interview was coded, and so, it is considered that the sample reached data saturation.

## Results

### Participants

Participants were HCPs (medical doctors, nurses, clinical psychologists, physiotherapists, and dietitians) from 13 NHS trusts (health care organizations) within England. Of the 26 participants, 8 (31%) were female. A total of 12 participants specialized in the care of patients with epilepsy, 6 in depression, 6 in MS, and 2 were general practitioners working across all 3 conditions. Participants included both primary and secondary care clinicians. Moreover, 13 of the 26 interviewees had used RMT with their patients, and 14 of the 26 said that their patients had brought data to appointments. The health care roles of the participants are presented in Table 1 along with their specialization and gender.

**Table 1:** Job roles, genders, and specializations of interview participants.

Clinical specialization		Depression (n=6)	Epilepsy (n=12)	Multiple sclerosis (n=6)	Generalist (n=2)	Total (N=26)
Gender (female), n (%)		1 (17)	4 (33)	2 (33)	1 (50)	8 (30)
**Job role**						
	Psychiatrist	4 (67)	0 (0)	0 (0)	0 (0)	4 (15)
	Psychologist	1 (17)	0 (0)	2 (33)	0 (0)	3 (12)
	Neurologist	0 (0)	6 (50)	3 (50)	0 (0)	9 (35)
	Dietician	0 (0)	1 (8)	0 (0)	0 (0)	1 (4)
	Specialist nurse	1 (17)	4 (33)	0 (0)	0 (0)	5 (19)
	Physiotherapist	0 (0)	0 (0)	1 (17)	0 (0)	1 (4)
	General practitioner	0 (0)	1 (8)	0 (0)	2 (100)	3 (12)

### Thematic Analysis

Our analysis generated 8 main themes, each of which also featured a number of subthemes.

#### Theme 1: Potential Clinical Value of the Remote Measurement Data

The interviews explored the types of physiological, psychosocial, and lifestyle variables that could be targets for measurement using RMT. The HCPs described uses for certain variables they considered to be potentially useful in the care of patients with 1 of the 3 conditions. Variables considered by participants to hold potential are summarized in Table 2.

**Table 2:** Target variables considered potentially useful to measure using remote measurement technology.

Condition	Variable
Epilepsy	Activity, anxiety, cognition, diet^a^, heart rate^b^, mood, quality of life, seizures (or proxies thereof), and sleep
Depression	Activity, anxiety, diet, mood, relapse signatures, sleep, and weight
Multiple sclerosis	Activity, anxiety, cognition, fatigue, mood, pain, quality of life, and visual acuity

^a^Opposing views on the value of measuring diet were offered by different participants.

^b^It was noted that heart rate would be worth measuring in epilepsy only if proven to be a proxy measure of seizures.

#### Epilepsy

Participants stated that it would be useful to collect data from RMT that could indicate the occurrence of a seizure or number of seizures, especially in those who have many. However, participants thought that the current approaches to seizure detection (eg, Empatica and Embrace) were limited by lack of sensitivity to detect the full range of seizure types:

The limitations of this particular device is that it is designed for detecting repetitive movements which is of use for tonic clonic seizures, however, there are different types of seizures which definitely don’t all involve movement but are still epileptic so they wouldn’t be able to detect that [...] It would be ideal to have something where all types of seizures would be recorded.

P02

Several types of RMT data were mentioned by participants as possible proxy measures of seizures. These were skin conductance, heart rate, accelerometry, pressure sensor readings, and electroencephalography. There were differing views on the usefulness of measuring heart rate and bed pressure as proxy measures for seizures, given the possibility of false positive signals (ie, lack of specificity).

Video and audio may be utilized remotely to assist with diagnosis and seizure identification, particularly through measurement of the sound and duration of a seizure:

I will sometimes tell people who struggle to video their loved one in a seizure for whatever reason, just to start the video going, put the phone down, and then it will both record the sound, and the duration of the seizure, which are probably the two most useful things we need, outside of seeing one.

P09

#### Depression

Activity data, including movement and GPS, were thought to be useful in detecting whether a patient was “leaving the house” [P21] or “getting out and about” [P06], which in turn could be considered a potential “proxy marker of depression severity” [P16]. This was also thought to have the potential to indicate a patient’s global level of functioning. RMT data were also thought to offer a level of objectivity in the measurement of depression, which was otherwise lacking. Prospective mood monitoring using an electronic diary and measurement of sleep using wearables were also mentioned as potentially helpful in managing depression, where currently, systems such as Fitbits and mood diary apps could not collate this information together automatically.

#### Multiple Sclerosis

Participants saw potential in the use of RMT to measure fatigue, via a self-report app, and also cognition, given its association with relapse. However, some considered it difficult to pick one particular aspect of MS to measure using RMT because the symptoms experienced by patients vary:

I don’t think you can have a particular tool that you would need to use for everyone. I think it is largely going to be dependent on the symptom profile.

P20

Multiple participants mentioned visual acuity as an important indicator of relapse, which could be measured using RMT, although they were unsure if technology would be able to measure it when even well-trained humans struggled:

It would have to be really well designed to pick up those intricacies [...] sometimes it is really difficult even for the neurologist to say this person is having a relapse or they are not.

P19

#### Theme 2: When to Use Remote Measurement Technology in the Care Pathway

**Figure 1:**
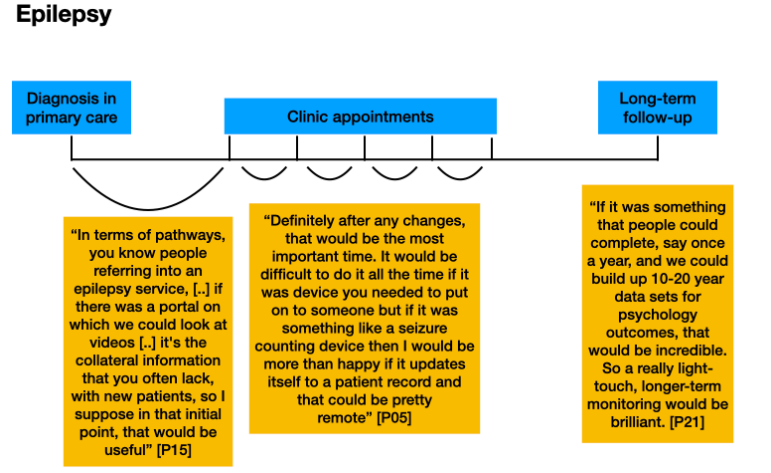
Participants’ comments about the timing of the use of remote measurement technology in the epilepsy care pathway, including monitoring on a yearly basis once stable to allow assessment of follow-up and to create data for future research.

**Figure 2:**
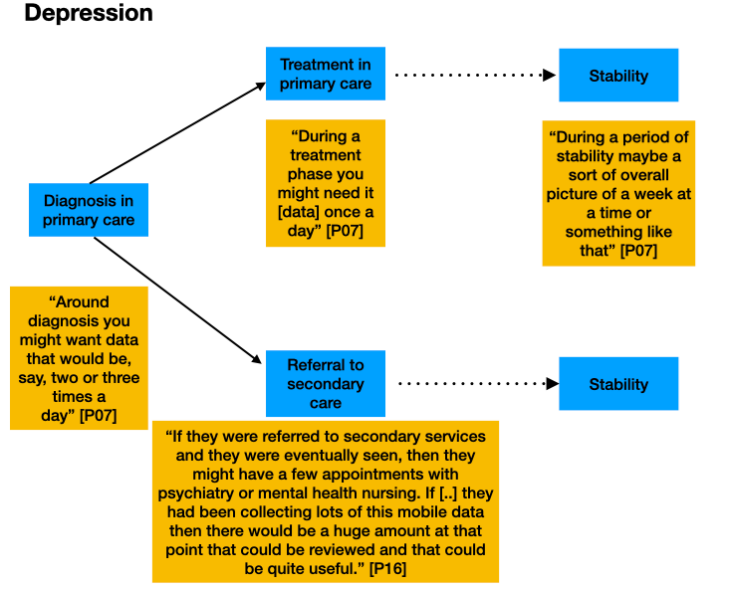
Participants’ comments about the timing of the use of remote measurement technology in the depression care pathway, including monitoring during a change in treatment.

**Figure 3:**
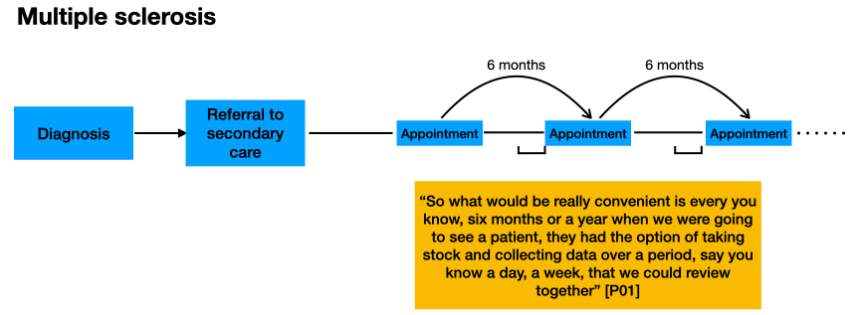
Participants’ comments about the timing of the use of remote measurement technology in the multiple sclerosis care pathway, indicating the benefits of monitoring for a short period before an appointment.

Participants described different points in care pathways when data from RMT could usefully inform the care of their patients. Figures 1-3 demonstrate where participants indicated that it could be useful to receive data from RMT. Clinicians indicated that they would like data collected via RMT to be readily accessible in their electronic patient records (EPRs) when patients attended for appointments.

It is acknowledged that not every patient’s journey along a care pathway follows the same trajectory. These figures are for illustrative purposes only.

#### Theme 3: Roles of Health Care Staff Who May Use Remote Measurement Technology Data.

Participants discussed the roles of health care staff who should be involved in using any RMT data. Across all 3 conditions, participants suggested that primary care was a good place for data to be managed, given the systems available:

Primary care is certainly quite well set up with systems in place to action on things based through the electronic patient record.

P06

In secondary care, specialist nurses were considered to have the closest relationships with patients among members of the care team, and thus, they were suggested to be the team member most likely to review data from RMT. For example, in epilepsy:

If it’s sort of data that is being in some way downloaded in between clinics, then there would certainly be a role for something like an epilepsy nurse to look at that data.

P09

Participants considered it important that all clinical, but not administrative, members of a team involved in the treatment of a patient should have access to RMT data when it is collected:

The discussions we’ve had are about, is it appropriate for admin staff to review that or actually does it have to be a clinician from a risk perspective [...] Your economic arguments come in about experienced clinicians are too expensive but I think making the wrong triage decision is also too expensive.

P21

#### Theme 4: Presentation and Accessibility of the Remote Measurement Technology Data

Participants had a variety of views on the best way for data to be presented to them. Particular emphasis was placed on the importance of interoperability and the ability for any data from RMT to be accessible within an existing EPR system rather than requiring the opening of another window or program:

If [...] you want to have the information available to you at times other than when your patient is there, then it would be good if it could feed directly into your electronic patient record.

P07

Across all 3 conditions, HCPs were keen to have data aggregated in a visual or graphical format. Some also recalled instances where they had used devices or software that presented information in graphs automatically and commented that this was useful for the patient as well as for the clinician:

For most of them [mood-reporting apps] you can do a graph function so they can show you the whole three months, in a fairly small chart, which helps us to think about if there have been any stresses or life events that have changed their mood or whether there’s a pattern to the time of day, and so on.

P07

The speed of access to information was also considered to be a priority. Participants spoke of particular situations in which the use of digital records could increase efficiency through time saving:

I certainly think if you can access the information quickly then it could be a focus point for the whole consultation and it could speed things up.

P16

Uploading data to the EPR was considered preferable to reviewing data that patients brought to the clinic on their mobile phones:

It could be sent in and loaded up in the patients notes or some other big screen device otherwise you are kind of stuck with little handheld mobiles and it’s not really that helpful.

P13

#### Theme 5: Obstacles to the Successful Use of Remote Measurement Technologies

Participants mentioned several aspects of the use of RMT, which they considered to be obstacles to their successful use and adoption in practice. These fitted into 2 broad categories of *technical issues* and *human issues*.

The most frequently mentioned technical issue was data accuracy (“I’m not sure they’re accurate*.*” [P03]). However, participants indicated that they would be happy to use devices even when they provided data that were not 100% accurate, so long as the clinician was aware of the margins of error that the data may contain:

So being as clear as possible what the potential pitfalls might be about all the data that we get back [...] I think as long as you know, kind of where it might go wrong, or how to be careful which bits to not over-interpret, then I think it’s fine

P09

There was also concern about the interoperability of any new system with existing ones, as clinicians are already required to use several different software packages to manage patients:

The main trouble currently is a lack of integration.

P01

Data security was also discussed as an important issue to consider, although participants had differing views on the level of risk that providing data remotely may entail. Some had concerns:

Who has access to this data? Including if they have it on their phone, what if their phone goes missing, where does this data go? There’s a big, data protection bit, there’s a big big, patient safety bit.

P03

Some advocated taking a pragmatic approach to reduce risk while continuing to use technology where it provided a benefit:

I think as long as appropriate safeguards are taken then that’s fine, and I think sometimes this can be a barrier, an unnecessary barrier to introducing things that can be helpful.

P06

Human issues considered obstacles to the use of RMT largely focused on 2 areas: patient anxiety and patient motivation. Participants discussed ways in which the use of RMT may cause patient anxiety through over focusing on their symptoms and how this in turn could be problematic for a health service:

We have had quite a few patients coming in that have used these monitoring devices and say my heart rate is really fast. For them it’s another layer of education so it actually creates us more work.

P03

However, others were less concerned, believing that RMT would not induce anxiety in patients who were not prone to it in the first place or that any anxiety would be manageable:

We’ve always found ways to react to that anxiety, this is just what it looks like in the current generation.

P21

Participants suggested that patients with depression would have less motivation to engage with RMT as a result of their condition:

A lot of our patients may, especially if they’re more severely depressed, not be very motivated to interact with the app

P16

However, the use of RMT to generate more objective evidence of a patient’s health state was considered by some to be useful in motivating engagement with their care:

So if you do your usual interview and you’ve got objective evidence to say, I think your depression is coming back or you haven’t been exercising enough or you have way more seizures than you think, then of course, that might help motivating them to do certain things.

P22

#### Theme 6: Limits to the Role of Remote Measurement Technology

Several of our participants mentioned elements of care in their specialism, which, in their view, should not be replaced by an RMT-enabled approach. In MS, the importance of face-to-face appointments was highlighted as essential for HCPs to identify subtle signs of worsening condition:

You really need to be physically examining the patient as well as hearing their perspective because there’s subtle deficits that you can pick up on at examination that people won’t notice day to day [...] you can pick up things like subtle signs like nystagmus, or problems with the balance or things like that, that people often won’t notice.

P01

In epilepsy too, HCPs reiterated the importance of seeing their patients face to face:

If their seizure frequency has increased you’re there thinking I probably need to see you, what else is going on? Have they got a cold, a water infection? Is there something else going on in their life? Are they not taking their tablets? Sometimes some of those conversations need to be had.

P03

A general practitioner mentioned the relational side of their work as important in the care of patients with depression:

The relationship element of it is very important, and obviously in primary care a lot of what I’m thinking is around depression [...] the human to human contact with someone who’s struggling with mood, and the fact that you’ve got someone who can be empathic and rapport rather than just crunching data.

P06

#### Theme 7: Empowering Patients

HCPs believed that RMT may benefit patients because it might empower them to take a leading role in their care. Some clinicians believe that patients should lead the use of technology and, therefore, have more control over their own care:

The way I see it is it’s more about the patient using the data for themselves, the clinician is almost the passive recipient of the data who is working with the patient to try and interpret it and help them develop techniques to use the data themselves.

P07

HCPs also spoke of how patients could be given full control of their own data collected using RMT and allow those data to be shared with a chosen clinician when they deemed it necessary:

Within the patient held database, [...] presumably a sort of secure log in, and that is, it’s patient-controlled [...] and they could give out the ability to share.

P15

Participants provided examples of how a patient could be empowered through the use of RMT, by determining when to arrange an appointment based on the outputs from the technology:

Through prospective mood monitoring you could capture periods where there had been a persistent lowering of mood over two weeks or more with associated other features or even shorter periods than that, that you’d agreed as part of a relapse signature. What people could do in those instances is potentially bring appointments forward.

P13

However, in the case of depression, participants saw difficulties with patient motivation and thus thought it would be unlikely that patients would be able to take control of their own care:

[That] involves them taking a lot of responsibility for their own healthcare and I guess that may work better in some conditions, more than depression.

P16

For some, it was a case of providing care on an individual basis:

I would tailor it to what they wanted, so you will have those who are very tech savvy who don’t have any time and think this will really suit me, others are very much I really want to see you doctor [...] the key is to listen to them and individualise care rather than doing tick box medicine which we sometimes do.

P03

#### Theme 8: Alert-Based Systems

There was debate across all 3 conditions in the interviews about the potential to use RMT to alert clinicians when a monitoring variable fell outside normative parameters, for example, if seizures increased in severity or frequency, if mood or activity were found to be particularly low, or if fatigue increased. The majority of participants considered such a system to be beneficial, so that interventions could be put in place as soon as possible:

It would be a system that had parameters set and triggered active alerts when those parameters were exceeded, I think would be the only way that I could see a lot of our consultant body engaging in it.

P01

However, a small number of participants thought that such a system would be problematic, principally because alerts may create excessive demand for immediate processing, interpretation, and response (eg, outside of normal working hours), and there would not be enough health care staff available to respond to the alerts produced:

Outside of fixed appointments the question would be who would actually have time and headspace to actually look at what was being flagged up. You would need to really carefully think about the staffing in the NHS and mental health services.

P13

HCPs also perceived there to be a risk that alerts would go unnoticed in the system:

My worry is this data arrives and nobody looks at it for weeks, it’s sitting somewhere in the ether.

P03

Several participants suggested that it would be more useful if the technology alerted the patient to take action through their regular treatment pathways, rather than putting the onus on the clinician:

I would want it to prompt the patient to make contact with me.

P14

Some saw a need for further research to determine the benefits of an alert-based system:

Unless you could do a good study and demonstrate that sending me alerts from an automated app would be helpful, then I would just want information that I could look back on when I next met with a patient face to face.

P14

## Discussion

### Principal Findings

A total of 8 themes emerged from the analysis of our interviews. The first theme covered the potential clinical value of the remote measurement data. Where RMT are currently used in health care practice, HCPs find them to be largely inaccurate, particularly in the case of epilepsy, although efforts to develop more effective ways of monitoring epilepsy are welcomed. Participants were optimistic about the future use of activity data to monitor symptoms of depression and considered that using RMT to collect measures of fatigue and cognition in patients with MS would be useful.

In theme 2, key points in care pathways for the 3 conditions were identified as times where RMT data could provide most value. These included monitoring a short period before an appointment (MS), monitoring during a change in treatment (epilepsy), and monitoring on a regular basis once a patient was in a stable condition, to allow assessment of follow-up and to create data for future research (depression, epilepsy, and MS).

The third theme considered staff roles in the management of RMT use by patients. Participants suggested that all staff involved in a patient’s care should have easy access to data generated by RMT via the patient record. Participants also made it clear that triage using data from RMT should be conducted by qualified HCPs rather than by administration staff. Primary care staff and specialist nurses in secondary care were thought to be well placed to manage incoming data from patients.

With regard to the presentation of data, in theme 4, HCPs described ease and speed of access to RMT data to be important for their successful use and emphasized the importance of interoperability with the patient record. Presentation of data in graphs was mentioned as helpful for interpretation.

Theme 5 discussed obstacles to the successful use of RMT, and these included both *technical issues* such as data accuracy and data security (where views differed on the risks involved) and *human issues* such as anxiety created by monitoring (although not all participants agreed that this was an issue).

In theme 6 on the limits of RMT benefit, participants emphasized that RMT would never completely replace face-to-face appointments, particularly in depression where relationships were considered important.

The seventh theme concerned patient empowerment. HCPs expressed the value in providing patients access to their own data, enabling them to take an active role in their own care, for example, by advancing appointments where RMT data indicated it was necessary. However, there was some concern about patients with depression having the motivation to take responsibility for their own care.

Theme 8 was related to alert-based systems. Participants debated the value of such systems and highlighted the requirements for their successful use. Some thought alerts should be used to invite the patient to take action rather than alerting a clinician, due to workload concerns. The need for further research to determine the benefits of alert-based systems was also highlighted.

### Comparison With Prior Work

Although prior work exploring RMT in health care has principally identified benefits and barriers to its implementation [11,14], this study has investigated HCPs’ perceptions of the clinical value of implementing RMT, helping to address the knowledge gap identified by Vegesna et al [13] and Davis et al [16].

The themes emerging in this study add to findings from prior work in this area. Our findings support the work of Bruno et al [18], who highlighted that HCPs may view the management of data from digital devices as a burden. Goodrich et al [19], among others, have highlighted the importance of interoperability and a preference for data from mobile technologies to be automatically integrated into clinical records, similar to the views of our participants. Clinicians’ concern about the need to respond to alert-based systems has also been raised previously [16,20].

Prior work has also emphasized the importance of face-to-face contact in the context of digital technology and mental health care [21]. A priority setting exercise for digital mental health [15] identified the need to explore the impact of removing such interactions from care pathways. Our data have shown that HCPs view face-to-face appointments as essential in the care of patients with these 3 conditions, even where RMT could provide them with detailed recent data on a patient’s status. Our data show that HCPs imagine patients could be empowered to determine their own need for a clinical appointment based on data from RMT, helping to address questions around the impact of technology on access to services, which has also been identified as a research priority [15].

Davis’ Technology Acceptance Model [22] describes perceived usefulness and perceived ease of use as key mediators to the successful uptake of a new technology. Our analysis highlights ways in which clinicians perceive RMT data could be useful (theme 1) as well as where there are limits or obstacles to this usefulness (themes 5 and 6). We have also identified how speed and ease of access to data are desirable for HCPs (theme 4), evidencing how perceived ease of use is applicable to this area. The analysis also raises the tailoring of care for patients using RMT (theme 7), where it was discussed that patients’ perception of RMT should be that it is both useful and easy to use to motivate continued use.

Beyond the findings presented in previous work, our findings specify the types of RMT data that clinicians would value in the management of epilepsy, depression, and MS as well as the points in patient care at which these data would be of most use and the health care roles that would be best placed to manage these data.

### Implications for Researchers and Developers

Findings from these themes will help to inform the development of the RADAR-CNS approach in the application of RMT for better care for epilepsy, MS, and depression. Researchers and companies developing monitoring technologies should ensure that the boundaries of accuracy of any new solution are well defined, such that clinicians can understand the level of confidence they should place in readings from such devices. As HCPs believe patients may benefit from the option to move or advance appointments based on their data, it would also be worthwhile for any mobile health solutions to link with appointment planning services, so that these can be easily accessed. In the United Kingdom, the NHS app is an example of such a system.

### Strengths and Limitations

This study has several strengths. We recruited a multidisciplinary group of HCPs working in a variety of clinical staff roles in primary and secondary care. Therefore, the use of RMT was considered from multiple perspectives. The study was limited in its consideration of only three specific central nervous system disorders in one national health care system. However, the analysis has considered how insights gained from staff working in these three conditions might generalize and has permitted a deeper analysis of the three conditions mentioned. The ratio of male to female participants was high, with only 8 of 26 participants being female. Epilepsy staff were over-represented in comparison with depression and MS staff due to the convenience sampling method. However, staff were represented across most roles in the care team for each of the three conditions represented, with the exception of MS, where an MS nurse could not be recruited in the time available.

### Future Directions

Although we have focused our consideration of the use of patient RMT data on an individual basis, further work could usefully explore the use of combined RMT data from groups of patients to assess risk or identify trends. The 2019 Topol Review highlighted the potential of integrating predictive analytics into diagnosis and care pathways [23], and data from RMT could feed into these approaches.

Future work should also explore the views of health service managers, commissioners, and public health representatives to understand the value that the implementation of RMT could provide from a health care system payer and management perspective, for example, in its potential to increase efficiencies and improve outcomes for different patient populations.

Given the participants’ views on the importance of nurses’ roles in the management of patients’ use of RMT, it would be useful to conduct further research to better understand nurses’ views on subsuming associated responsibilities into their roles. Although some work has explored nurses’ views on their roles in the use of technology in intensive care situations [24] and telehealth for diabetes [25], to our knowledge, no studies have explored views specifically relating to RMT in central nervous system disorders.

Further work should also be completed to understand how RMT might best facilitate increased patient autonomy (as advocated in the NHS Long Term Plan [26]) and situations where this may be less appropriate or successful. The remote assessment of disease and relapse-major depressive disorder study is recruiting 600 people with a major depressive disorder to use RMT over a period of 2 years, and this study may shed light in this area [27].

### Conclusions

This paper has explored the views of HCPs on using RMT in managing central nervous system disorders, specifically epilepsy, MS, and depression. The results are as follows:

- target physiological variables for measurement that clinicians believe would be useful
- points in care pathways at which clinicians perceive benefit to patients using RMT
- roles of health care staff best placed to manage incoming data
- HCPs’ preferred presentation of data
- obstacles to the successful implementation of RMT
- limits to the benefits that the RMT can provide
- ways in which patients may be empowered through the use of RMT
- considerations around alert-based systems.

Our findings show the importance of early engagement and co-design with HCPs when considering user requirements and potential use cases before using RMT in clinical care pathways. HCPs believe that RMT data can add value to the care of patients with these three conditions but are not sufficient for decisions about care to be made exclusively on the basis of these data. We have demonstrated that clinicians are pragmatic about the data security risks of using RMT data with patients. Further research is required to establish how RMT data could be used on a population level to benefit patients with central nervous system disorders.

## Abbreviations

EFPIA: European Federation of Pharmaceutical Industries and Associations
EPR: electronic patient record
HCP: health care professional
MS: multiple sclerosis
NHS: National Health Service
NIHR: National Institute for Health Research
RADAR-CNS: Remote Assessment of Disease and Relapse-Central Nervous System
RMT: remote measurement technology
